# A Six-Quadrupole Diamagnetic Suspension Architecture for Ultra-Sensitive Space Accelerometry

**DOI:** 10.3390/s26206640

**Published:** 2026-10-20

**Authors:** Marco Pisani, Edoardo Dalla Ricca, Carlo Paolo Sasso, Massimo Zucco

**Affiliations:** Istituto Nazionale di Ricerca Metrologica, Strada delle Cacce, 91, 10135 Torino, Italy; e.dallaricca@inrim.it (E.D.R.); c.sasso@inrim.it (C.P.S.); m.zucco@inrim.it (M.Z.)

**Keywords:** space accelerometer, diamagnetic suspension

## Abstract

This paper proposes a novel inertial sensor based on a solid diamagnetic cubic test mass passively suspended by a symmetric configuration of six magnetic quadrupoles. Designed specifically for microgravity environments, this system eliminates the mechanical noise and hysteresis inherent in elastic suspensions, as well as the complexity associated with electrostatic suspension systems. We provide an analysis of the magnetic intrinsic noise floor imposed by eddy current damping, alongside ground-based preliminary experimental results demonstrating potential sensitivities in the order of the $10^{-10}\;{m/s}^{2}/\sqrt{\mathrm{Hz}}$ regime.

## 1. Introduction

Acceleration measurements play a crucial role in a wide range of space applications, ranging from fundamental physics experiments, to satellite geodesy and accurate orbit determination and correction for navigation satellites [1,2,3,4]. In a microgravity environment, spaceborne accelerometers operate by measuring the relative displacement of a nominally free-floating test mass with respect to its spacecraft housing. Non-inertial accelerations acting on the spacecraft, such as those arising from solar radiation pressure or residual atmospheric drag, are imparted to the spacecraft, while the test mass remains in near-free fall. The resulting differential motion therefore produces a measurable displacement between the test mass and its surrounding housing. To realize this measurement in practice, the test mass must be physically or virtually constrained to remain within a defined working volume. Consequently, the core architecture of any space accelerometer is defined by two essential elements: the constraint (suspension) system and the displacement readout system.

The simplest form of suspension is a mechanical elastic element (spring), characterized by a specific stiffness constant *k*. Depending on the suspension geometry, the test mass may remain free to move along between one and six degrees of freedom (DoFs). For example, a blade spring constrains all but one translational DoF, resulting in a single-axis accelerometer (such as ISA aboard BepiColombo [5]), whereas a coil spring can provide compliance in all six DoFs, each characterized by a different effective stiffness. Regardless of the implementation, the mechanical sensitivity of a spring–mass accelerometer, defined as the displacement produced per unit applied acceleration, is fundamentally determined by the restoring stiffness and the proof mass. When operated well below the mechanical resonance, the proof-mass displacement is as follows:

(1) $$x=a/\omega_{0}^{2}$$

where *a* is the applied acceleration and $\omega_{0}$ is the natural angular frequency of the test mass. Therefore, for a given mass, maximizing sensitivity requires minimizing the spring constant *k*.

To achieve this ultra-low stiffness, state-of-the-art space accelerometers predominantly utilize electrostatic suspensions [6,7]. Through active control loops, the electrostatic forces between the test mass and fixed electrodes mounted on its surrounding housing are controlled to create a virtual spring, allowing the stiffness to be reduced to extremely low values. While highly effective, the main disadvantage of electrostatic suspensions is the strict requirement for a continuous, closed-loop active control system, which relies on the knowledge of the test mass position within its housing.

Meeting this requirement places stringent demands on the readout system. Capacitive sensing is the most widely adopted solution, in which the position of the test mass is inferred from changes in the capacitance between the test mass and the surrounding electrodes [8]. Alternatively, optical interferometric readouts have demonstrated even higher displacement sensitivities [9,10].

In this paper, we introduce a novel paradigm for test mass constraint systems: a fully passive, contactless architecture based on the magnetic suspension of a diamagnetic test mass within a system of magnetic quadrupoles. By exploiting the large diamagnetic susceptibility of solid bismuth and the high degree of symmetry provided by the magnetic housing, we demonstrate the feasibility of generating a stable three-dimensional magnetic potential well. This approach eliminates mechanical dissipation and noise associated with conventional spring-based suspensions, and removes the complexity of active feedback schemes typically required in electrostatic sensing systems. The resulting architecture provides a simple solution for the development of next-generation ultra-sensitive space accelerometers.

The use of diamagnetism for the levitation of small masses and for the development of tilt and acceleration sensors has been previously demonstrated, but primarily in the presence of gravity, which provides a natural mechanical constraint, and for very small test masses. Moreover, none of these approaches has employed a symmetric three-degree-of-freedom (3-DoF) structure [11,12,13,14,15].

## 2. Working Principle, Architecture and Gap-Tuning

The core of the sensor consists of a cubic bismuth test mass enclosed within a three-dimensional magnetic trap. The test mass is suspended inside a housing formed by a point (central) symmetric configuration of six magnetic quadrupoles, providing contactless confinement along all three spatial axes.

### 2.1. Magnetic Configuration

**Figure 1:**
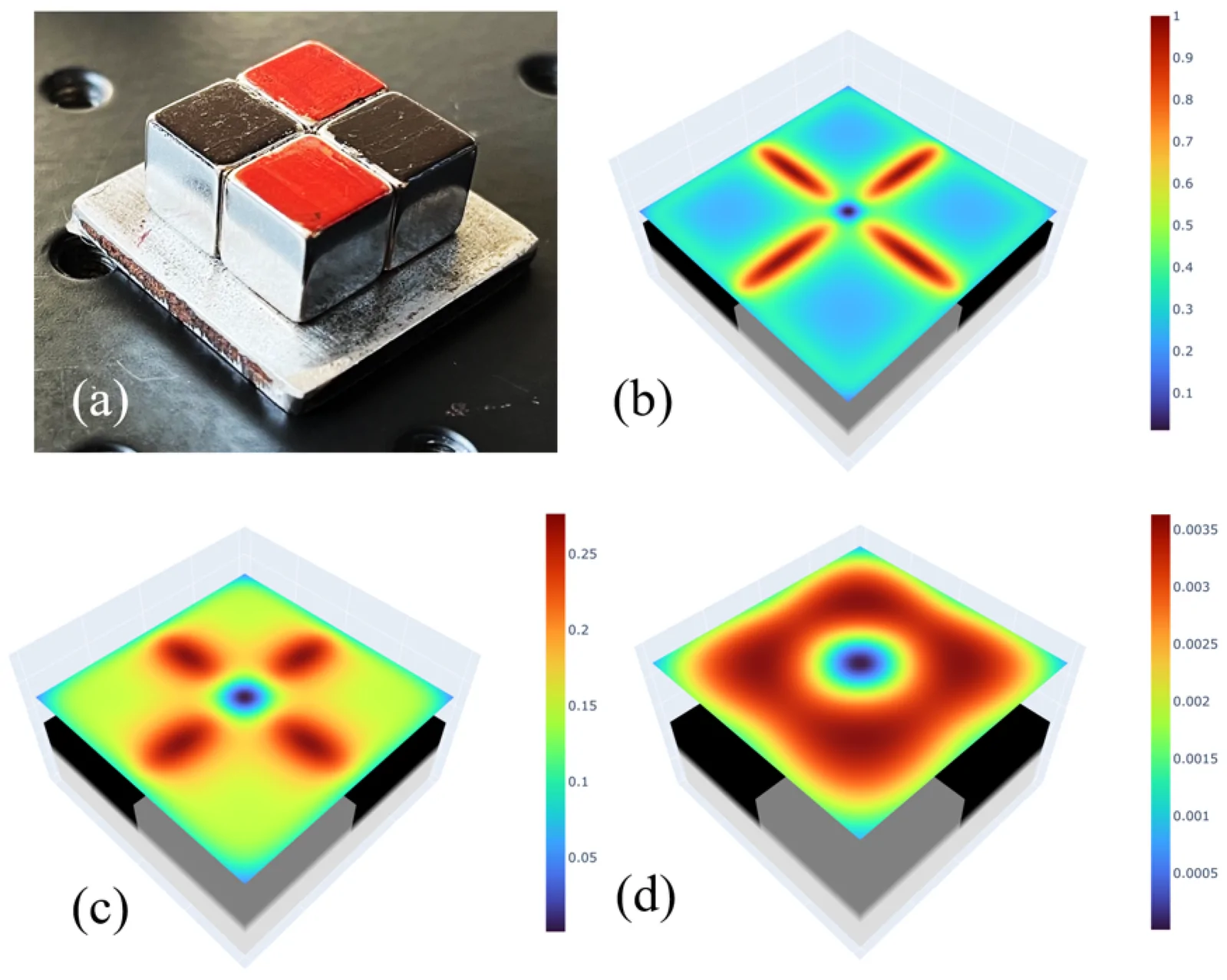
(**a**): Picture of the single quadrupole element. The poles are colored for clarity; (**b**–**d**): graphical representation of the value of B∇B for different distances from the quadrupole surface: respectively 1 mm, 2 mm and 4 mm. The false color scale is normalized to the maximum value of the 1 mm case.

As a baseline for the following analysis we have considered a cubic test mass having *l* = 25 mm and *m* = 153 g. Each quadrupole is composed of four cubic NdFeB permanent magnets (10 mm side length) arranged in a checkerboard magnetization pattern (N–S/S–N), as shown in Figure 1a. To enhance the inward concentration of the magnetic flux, the rear face of each quadrupole is backed by a 30 × 30 × 2 mm soft iron plate which has also the purpose of shielding the magnetic flux outwards. This configuration generates a theoretical zero-field point at the geometric center of the trap, surrounded by a region of extremely large magnetic field gradient (∇B). Since the diamagnetic force is proportional to the product of the magnetic field and its spatial gradient (F∝B∇B), the bismuth test mass experiences a repulsive force from each quadrupole (see Figure 1b–d). The arrangement of six quadrupoles will act as a multidimensional virtual spring, or 3-dimensional potential well, pushing the mass towards the center of the housing.

### 2.2. Gap Tuning

The gap is defined as the clearance between the surface of the test mass and the magnetic poles. We have taken as a baseline a nominal gap of 2 mm, achieving a compromise between restoring force and allowable displacement. Because the magnetic field generated by the quadrupoles decays rapidly with distance, increasing the gap results in a significant reduction in both the restoring force and the effective magnetic spring stiffness. An advantage of this configuration over conventional mechanical springs is the ability to tune the system stiffness by varying the gap between the levitated mass and the surrounding structure. Reducing the stiffness correspondingly lowers the resonance frequency, potentially bringing it into the mHz regime thus enhancing the accelerometer’s sensitivity in a controllable manner. Moreover, as discussed in the following section, increasing the gap also lowers the instrument’s intrinsic thermal-magnetic noise floor.

## 3. Fundamental Noise Analysis and Bismuth Anisotropy

The ultimate resolution limit of the accelerometer is governed by the Fluctuation-Dissipation Theorem (FDT) [16] of electromagnetic nature, given that mechanical linkages are not present. The movement of the conductive bismuth mass through the intense magnetic gradient induces eddy currents within its volume. These currents dissipate energy via Joule heating, introducing a magnetic damping coefficient $\Gamma_{mag}$. This dissipation is inextricably linked to a stochastic dissipative force noise, with an amplitude spectral density of acceleration noise as follows:

(2) $$S_{a,th}^{1/2}=\sqrt{\frac{4k_{B}T\Gamma_{mag}}{m^{2}}}$$

where $k_{B}$ is the Boltzmann constant, *T* is the thermodynamic temperature and *m* is the mass of the test mass.

The power dissipated by eddy currents scales with the square of the magnetic-field spatial gradient, consequently, the associated thermal acceleration noise scales linearly with the magnetic-field gradient. The acceleration noise is obtained by first evaluating the viscous damping due to eddy currents induced in the bismuth cube as it moves through the magnetic-field gradients, which reaches approximately 75 T/m for a 2 mm gap and 40 T/m for a 4 mm gap. The resulting total damping coefficients are $\Gamma_{mag}\approx 0.133\;N\;s/m$ and $\Gamma_{mag}\approx 0.037\;N\;s/m$, respectively. Applying the Fluctuation–Dissipation Theorem at T=300 K, these damping coefficients yield thermal acceleration-noise spectral densities of

(3) $$S_{a,th}^{1/2}\approx 3.07\times 10^{-10}\;m/s^{2}/\sqrt{\mathrm{Hz}}$$

for the 2 mm gap, and

(4) $$S_{a,th}^{1/2}\approx 1.61\times 10^{-10}\;m/s^{2}/\sqrt{\mathrm{Hz}}$$

for the 4 mm gap. Increasing the gap from 2 mm to 4 mm approximately halves the magnetic field gradient, reducing the thermal magnetic noise floor by a factor of two. It can be noticed that since the damping coefficient in Equation (2) is dependent on the strength of the magnetic field, the noise is also dependent on the position of the mass with respect to the magnets; in fact we have calculated the spectral densities in (3) and (4) for the exact equilibrium position of the test mass, assuming that this is the preferred working position.

Beyond this, the strong anisotropy of the diamagnetic susceptibility of the bismuth crystal is a critical property to be addressed [17]. If the test mass consists of large single crystals, this anisotropy results in asymmetric restoring forces within the otherwise symmetric magnetic housing. To ensure an isotropic macroscopic magnetic response, the formation of large crystals must therefore be suppressed. Manufacturing techniques such as rapid solidification or mechanical agitation during casting promote the formation of a fine-grained polycrystalline microstructure with randomly oriented crystallites, effectively averaging out the intrinsic anisotropy.

On the other hand, the intrinsic thermal-magnetic noise can be reduced by replacing the solid crystalline test mass with a sectorized structure (such as smaller cubes separated by dielectric layers), to reduce eddy currents. An alternative and potentially optimal approach is to employ powder metallurgy, in which the bismuth is sintered from electrically isolated grains. This approach offers the possibility of addressing both limitations simultaneously: a test mass fabricated from pressed, randomly oriented bismuth particles with insulating coatings would provide near-isotropic magnetic properties while substantially increasing the macroscopic electrical resistivity thus reducing the eddy current damping at the material level.

## 4. Experimental Setup and Preliminary Results

**Figure 2:**
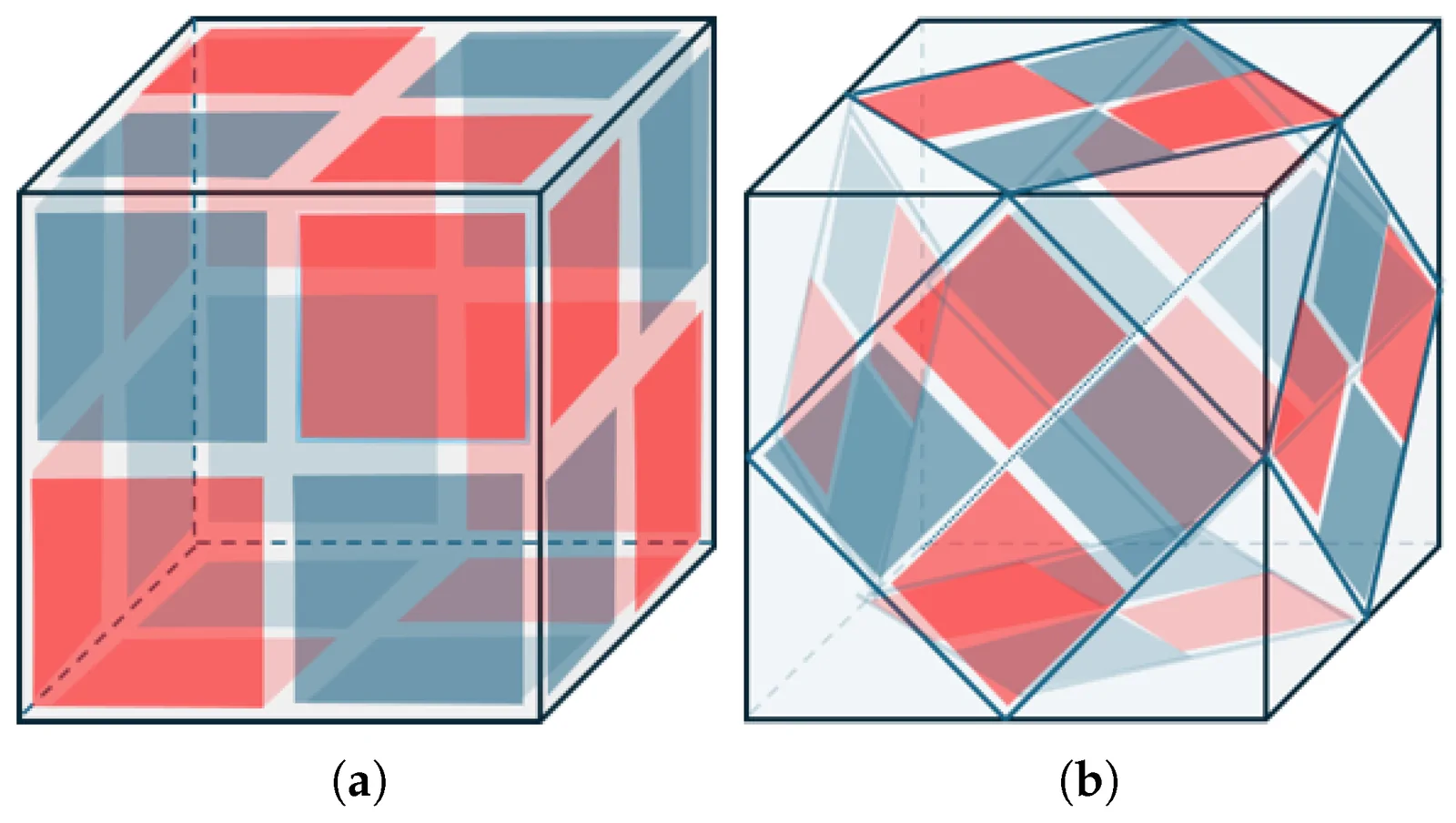
The two proposed point symmetric configurations. (**a**): the axes of the quadrupoles are parallel to the test mass edges; (**b**): the axes of the quadrupoles are rotated by 45° with respect to the mass edges. Note the different arrangement of the magnets in the two configurations to maintain the full symmetry. On each face, the red and blue squares represent permanent magnets with alternating magnetization directions (toward and away from the face).Also note that the purpose of these illustrations is to show the relative orientation of the quadrupoles on the six sides for the two configurations; therefore, the proportions of the drawings are not relevant.

**Figure 3:**
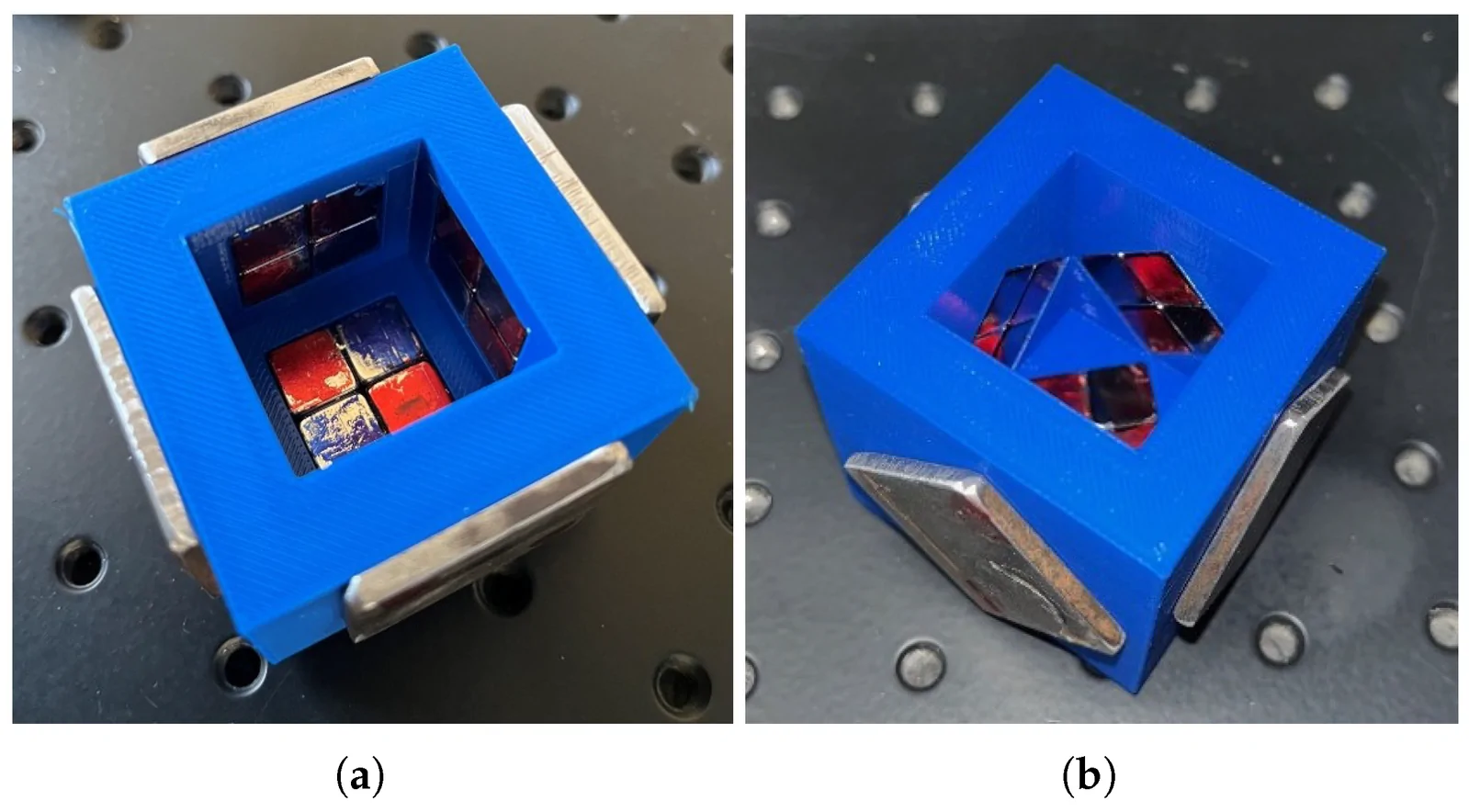
Experimental implementation of the straight five-sided housing (**a**) and the 45° five-sided housing (**b**).

Two different configurations of the checkerboard magnetic pattern have been considered. In the first configuration (Figure 2a), the pattern is aligned with the cube’s housing edges, whereas in the second (Figure 2b), it is rotated by 45°. Note that in the 45° configuration, each blue vertex connects to a red vertex, resulting in a perfectly symmetric configuration. The same approach is not possible with the straight quadrupole configuration, for which the only possible arrangement is to have red vertices connected to red vertices and blue vertices connected to blue vertices. The experimental results presented in the following sections are obtained using the first configuration; however, comparable results are also obtained with the 45° configuration, demonstrating that both configurations are effective in trapping the test mass. More detailed experimental investigations or finite-element analysis may reveal differences in the trapping efficacy of the two configurations. We intend to investigate these aspects in a subsequent, more quantitative experimental study. Figure 3 shows the practical implementation of the magnetic trap realized with a plastic structure embedding five elementary quadrupole units shown in Figure 1a.

A ground-based experimental campaign is carried out using a simple pendulum testbed to validate and demonstrate the trapping concept. The suspension system consists of a 116 cm long 1K carbon-fiber wire supporting a solid bismuth parallelepiped with a square cross-section (25×25 mm^2^) and a height of approximately 30 mm, resulting in a test mass of about 180 g. The moment of inertia of the suspended mass about the rotation axis is $I_{z}=1.91\times 10^{-5}\;\mathrm{kg}\;m^{2}$. The pendulum motion is monitored using a Precitec optical displacement sensor (model “CROcodile2-S” equipped with optical head “Messkopf 10 mm, range 10 mm and resolution 0.1 μ m), enabling accurate measurements of both translational displacements and angular oscillations. Being the Precitec a 1D sensor, it was not possible to measure displacements and rotations at the same time. For the displacement measurements the optical beam was focused on a small target placed close to the hanging point of the mass in order to minimize the sensitivity to the residual rotations, while for the torsional measurements, the optical beam was focussed on one side of the cubic mass close to the edge, so to maximize the sensitivity to rotations. The pendulum configuration provides a 3 DoF system, in which the two horizontal translational degrees of freedom are counteracted by the gravitational restoring force, while rotation about the vertical axis is constrained by the torsional restoring torque of the suspension wire. The measured natural frequency of the pendulum modes is 0.47 Hz, while the torsional natural frequency is approximately 0.01 Hz, with both frequencies measured using the Precitec sensor.

**Figure 4:**
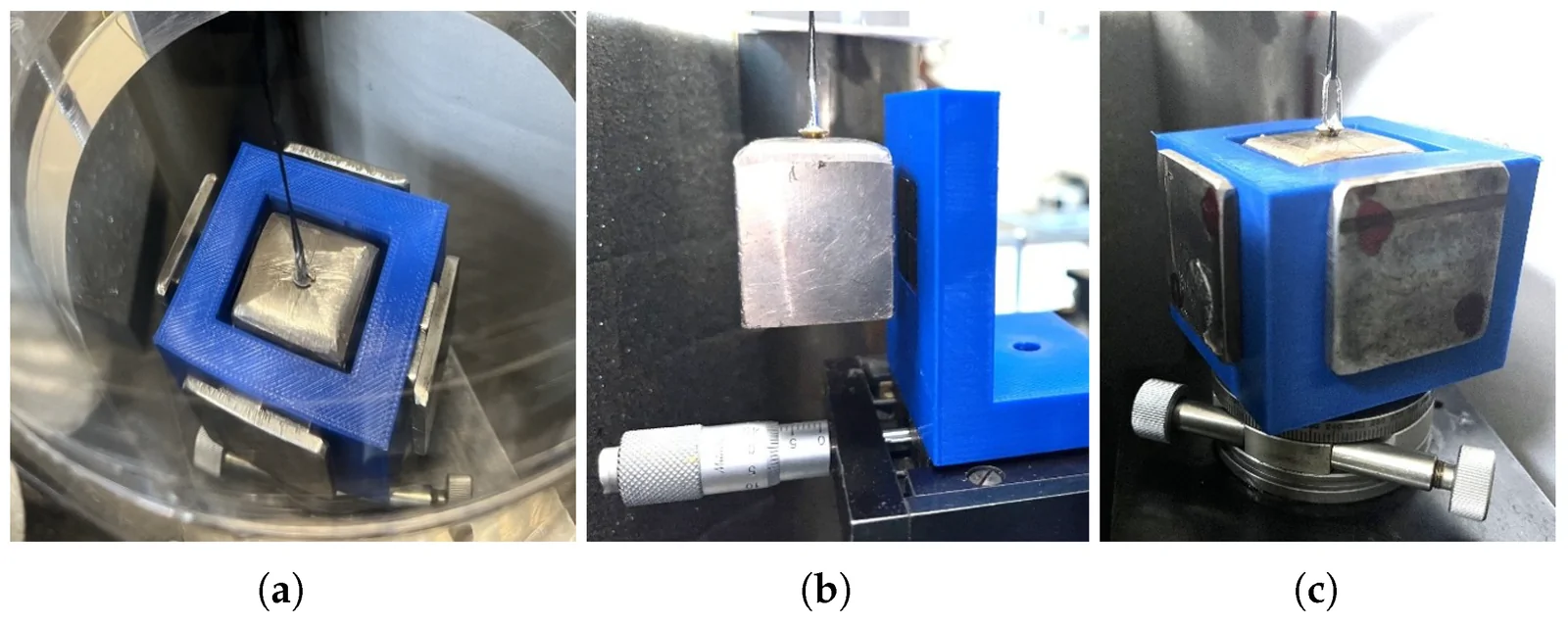
Pictures of the experimental setups. (**a**): suspended mass in the 5-sided housing seen from above where the gap is clearly visible. (**b**): experimental set-up for the measurement of the repulsion force where a single quadrupole is faced to the mass. (**c**): the housing placed on the rotation stage for the measurement of the torque.

Two key experiments are performed: the first to characterize the repulsive force and estimate the resulting translational trapping force, and the second to characterize the magnetic torque responsible for maintaining the alignment of the test mass within the trap. Figure 4 illustrates the different stages of the experimental procedures described below.

### 4.1. Repulsion Force and Translational Trapping

**Figure 5:**
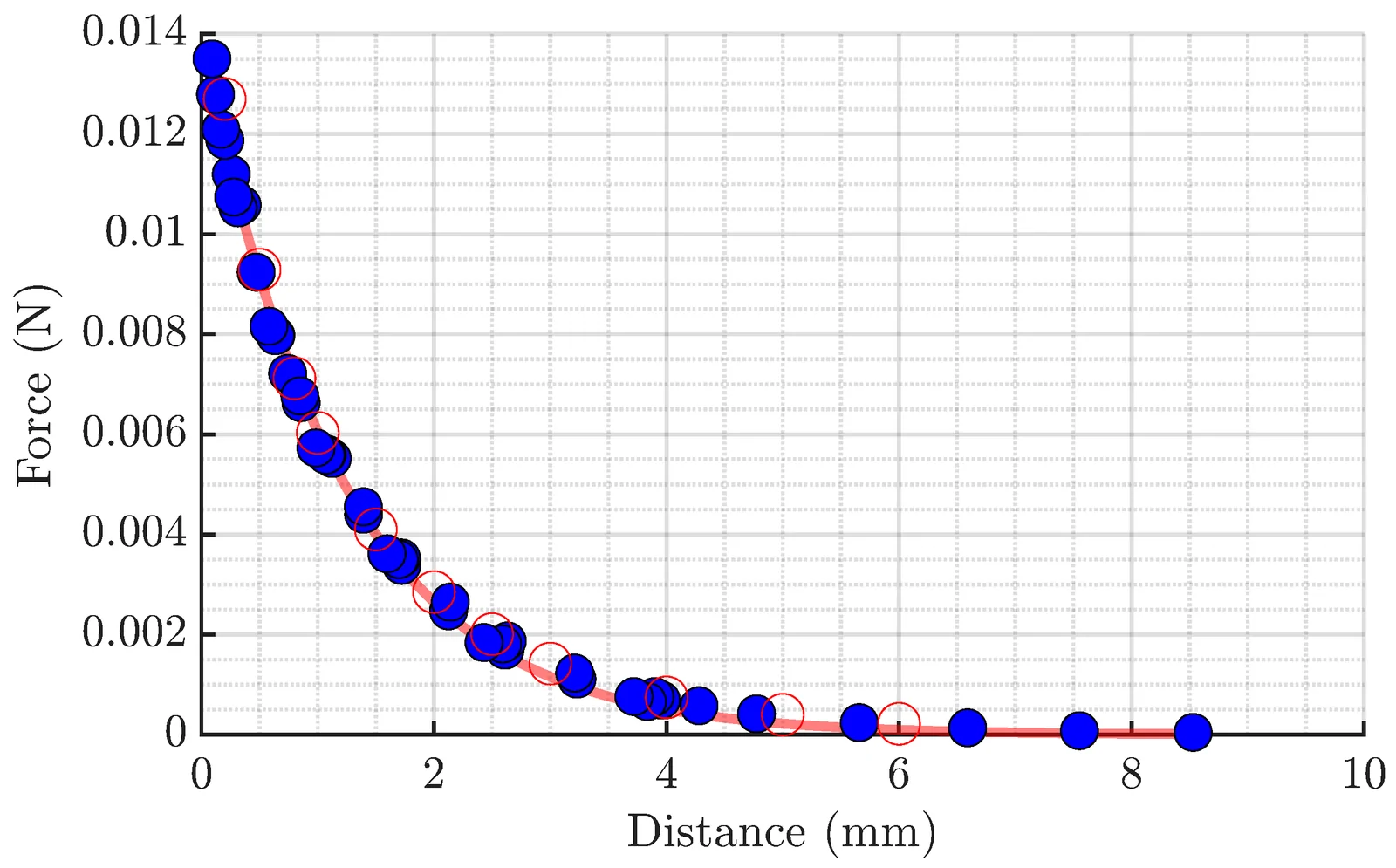
Plot of the repulsive force as a function of distance as generated by a single quadrupole acting on the test mass. The blue dots represent the measured experimental values; the empty red dots represent the predicted force according to the FEM model; the red curve represents the exponential fit of the measured data.

To measure the repulsive force as a function of the gap between the quadrupole and the test mass, a single quadrupole is mounted vertically on a translation stage driven by a micrometric screw (Figure 4b). The quadrupole faces one of the vertical sides of the test mass whose displacement is measured by the Precitec sensor positioned on the opposite side of the mass (not visible in the picture). As the quadrupole is translated toward the mass, the repulsive interaction causes the mass to move away in the same direction. The gap is determined from the difference between the quadrupole displacement measured by the micrometric screw and the displacement of the mass measured by the Precitec sensor. The resulting displacement of the mass from its vertical equilibrium position is then used to determine the repulsive force from the horizontal component of the gravitational restoring force. The results of multiple measurements are reported in Figure 5. In the force–distance plot, the blue markers represent the experimental measurements, while the red curve corresponds to an exponential fit to the data. The fit is described by the function $f\left(x\right)=ae^{bx}$, with fitting parameters a=0.014 and b=−0.83. The fitting curve is used to estimate the translational trapping force produced by a pair of opposing quadrupoles. The same experiment carried out with the 45° rotated quadrupole gave substantially the same results.

**Figure 6:**
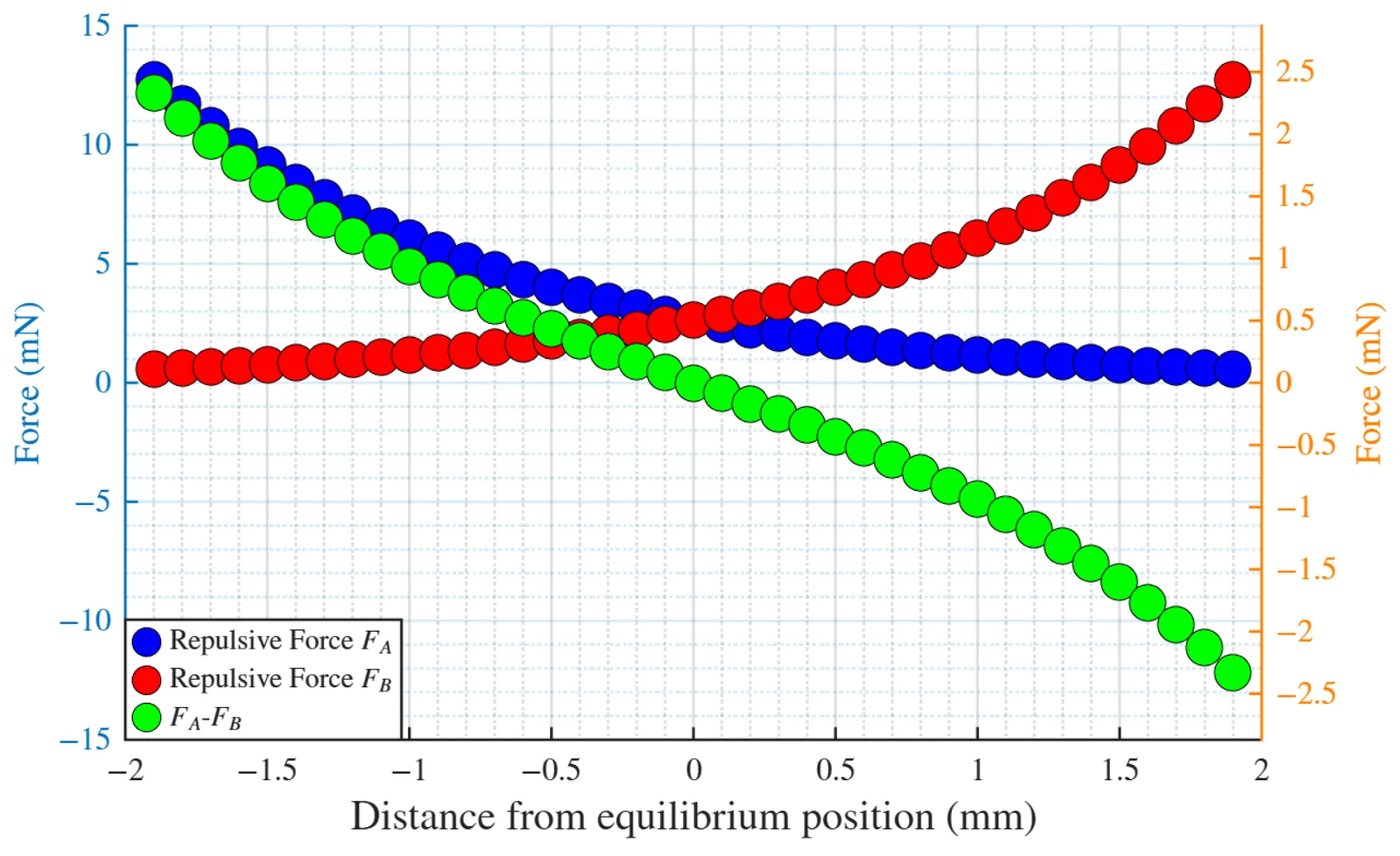
Trapping force generated by a pair of opposite quadrupoles, denoted as *A* and *B*, calculated from the exponential curve shown in Figure 5. The left and right vertical axes correspond to gaps of 2 mm and 4 mm, respectively. The horizontal axis represents the distance from the equilibrium (central) position of the test mass. The blue and red curves show the repulsive forces generated by quadrupoles *A* and *B*, respectively, while the green curve represents the resulting trapping force acting on the test mass, obtained as the difference between the two repulsive forces.

The trapping of the test mass is in fact generated by the repulsive forces from the quadrupoles on both sides, which balance each other at the central position. The analytical function obtained by fitting the experimental results has been used to estimate theoretically the trapping force. We have calculated the forces exerted by the two opposite quadrupoles and we have assumed that the resulting force is the linear sum (with opposite sign) of the two. The results are shown in Figure 6 where the net force acting on the test mass is plotted as a function of the displacement from the central equilibrium position. The figures have been calculated for the 2 mm and 4 mm gaps. At the central position, the net force is zero, and the force–displacement relationship can be locally approximated as linear, corresponding to an ideal spring with an effective spring constant *k* given by the derivative of the force with respect to displacement at the equilibrium position. The resulting spring constants are k=4.38 N/m and k=0.84 N/m for the 2 mm and 4 mm gaps, respectively. In this estimation of the translational trapping force, the contributions from the other four quadrupoles, which move parallel to the faces of the cube, are neglected, as they are expected to be significantly smaller.

To support the experimental results the open-source finite-element-method (FEM) multiphysics solver Elmer [18] is employed to calculate the repulsive force exerted by the magnetic quadrupole on the test mass. At this stage, the geometrical model consists of a single quadrupole from four cubic NdFeB permanent magnets, assembled in a straight chessboard pattern, and a cubic bismuth test mass. The geometrical parameters are the same used in the experimental set-up. The computational mesh is generated using Gmsh. The relative magnetic permeability of the homogeneous polycrystalline Bi test mass is set to $\mu_{r}=0.99984$, corresponding to a volume magnetic susceptibility of $\chi_{v}=-1.6\times 10^{-4}$. The FEM results reproduce the experimentally observed dependence of the repulsive force on the test-mass position using a single adjustable parameter, namely the remanent flux density of the permanent magnets. This parameter is set to $B_{r}=1.19$ T, corresponding to a magnetization of approximately 950 kA/m, consistent with the properties expected for the commercial NdFeB magnets used in the experiment. Figure 5 compares the simulated force values with the experimental data.

### 4.2. Torque and Rotational Trapping

Starting from the moment of inertia of the test mass and measuring the natural torsional period of the suspended mass (approximately 100 s), the torsional spring constant of the suspension wire can be estimated as $\kappa \approx 9.2\times 10^{-8}$ N m/rad. As discussed below, this contribution is negligible compared with the magnetically induced restoring torque. The test mass is then inserted into the five-sided quadrupole housing, leaving the top open to accommodate the suspension wire. The housing is mounted on a rotation stage, as shown in Figure 4c. When the housing is rotated, the test mass follows the rotation while maintaining its faces parallel to the housing walls, indicating the presence of a restoring magnetic torque. In order to quantify the value of the torque, a rapid angular displacement was manually applied to the housing, causing the test mass to oscillate about its axis without contacting the housing walls. The measured torsional natural frequencies are $f_{2mm}=0.77$ Hz and $f_{4mm}=0.4$ Hz for gaps of 2 mm and 4 mm, respectively. These measurements allow the magnetically induced restoring torque to be estimated for the two configurations as $\kappa_{2mm}\approx 4.46\times 10^{-4}$ N m/rad and $\kappa_{4mm}\approx 1.21\times 10^{-4}$ N m/rad for gaps of 2 mm and 4 mm, respectively. Both values are orders of magnitude larger than the torque of the wire, which, therefore, has been neglected.

### 4.3. Provisional Sensitivity Budget

Based on the results obtained above, we can theoretically estimate a preliminary sensitivity budget for an accelerometer based on the proposed magnetic-trap principle. The acceleration sensitivity is determined by the resonant frequency of the mass–spring system as in Equation (1), combined with the noise floor of the displacement readout. Note that here we are assuming that the frequency working range is lower than the resonant frequency of the system that is where Equation (1) is valid. Assuming a displacement readout noise density of 100 pm/Hz, which can be readily achieved using either interferometric or capacitive sensing [19], the corresponding acceleration noise density can be estimated and combined with the thermal noise contribution as obtained in Formulas (3) and (4). The resulting performance estimates for the proposed configuration are summarized in Table 1 and provide a baseline for further developments. Furthermore, it must be noted that this theoretical estimation does not take into account low-frequency noise sources such as random walk or flicker, typically due to correlated effects such as temperature drifts, mechanical creep, excess electronic noise, etc. These noises are typically dominant at low frequencies, but are difficult to be estimated theoretically, thus we intend to characterize the real low frequency performances through a dedicated experimental campaign. The present analysis shows that the readout noise is the dominant contribution over the thermal (damping) noise. This is expected to change in high-end applications, where sub-picometer displacement readout can be employed, potentially enabling acceleration noise levels approaching $10^{-13}\;{m/s}^{2}/\sqrt{\mathrm{Hz}}$ [9,10].

These preliminary results set the magnetically suspended accelerometer in a good position compared with presently available high-end accelerometers [4,5].

## 5. Conclusions

The proposed 6-quadrupole architecture is shown to constitute an effective passive alternative to mechanical and electrostatic suspensions, providing a stable and tunable magnetic potential well for the confinement of diamagnetic test masses. Ground-based pendulum measurements performed along three degrees of freedom (two translational and one rotational) demonstrate that the bismuth parallelepiped is stably trapped within the horizontal plane and naturally self-aligns with the walls of the magnetic system. Given the full symmetry of the 6-quadrupole configuration, the stability observed across these three degrees of freedom is expected to extend to the complete six degrees of freedom of a fully enclosed six-sided housing under microgravity conditions. The experimental results are in good agreement with the theoretical predictions obtained from numerical simulations developed in parallel.

Future work will address the following developments: the fabrication of a prototype with well defined geometrical parameters and a microcrystalline internal structure, aimed at mitigating anisotropy-induced effects; the repetition of the measurements under vacuum conditions to assess the damping coefficient; the realization of a full six-quadrupole assembly to be characterized in microgravity, for instance at the Einstein Elevator facility [20]; the implementation of an interferometric readout system capable of resolving picometer-level displacements and enabling a quantitative assessment of damping-induced noise as well as unpredictable low frequency noises; and the production of sintered or sectorized bismuth test masses designed to minimize thermal-magnetic damping. Taken together, these optimizations will pave the way toward the development of a flight-ready prototype. Further developments foresee the miniaturization of the magnetic trap to be compliant with the nano-sat market as well as the test of other diamagnetic materials such as highly oriented pyrolytic graphite (HOPG).

## Figures and Tables

**Table 1:** Parameter values and noise sources for gap = 2 mm and gap = 4 mm.

Parameter or Noise Source	Gap = 2 mm	Gap = 4 mm	Unit
Translational magnetic stiffness: $k_{\mathrm{mag}}$	4.38	0.84	N/m
Translational natural frequency	0.78	0.34	Hz
Torsional magnetic stiffness: $\kappa_{\mathrm{mag}}$	$4.46\times 10^{-4}$	$1.21\times 10^{-4}$	N·m/rad
Torsional natural frequency	0.77	0.4	Hz
Equivalent acceleration noise (readout noise $=10^{-10}$ m/$\sqrt{\mathrm{Hz}}$)	$2.39\times 10^{-9}$	$4.58\times 10^{-10}$	m/s^2^/$\sqrt{\mathrm{Hz}}$
Magnetic damping noise	$3.07\times 10^{-10}$	$1.61\times 10^{-10}$	m/s^2^/$\sqrt{\mathrm{Hz}}$
**Total acceleration noise**	$2.41\times 10^{-9}$	$4.85\times 10^{-10}$	**m/s^2^** **/** $\sqrt{\mathrm{Hz}}$

## Data Availability

Dataset available on request from the authors.
